# Comparison of Fasting Insulin Level, Homeostatic Model of Insulin Resistance, and Lipid Levels between Patients with Primary Hypertension and Normotensive Subjects

**DOI:** 10.5041/RMMJ.10468

**Published:** 2022-04-26

**Authors:** Rithvik Ramesh, Viswanathan Pandurangan, Sudha Madhavan, Devasena Srinivasan, Emmanuel Bhaskar, Lakshmi Marappa, Aiswarya M. Nair, Vaasanthi Rajendran, Priyadarshini Varadaraj

**Affiliations:** Department of General Medicine, Sri Ramachandra Medical College and Research Institute (SRMC&RI), Porur, Chennai 600116, India

**Keywords:** Fasting insulin level, HOMA-IR, hyperinsulinemia, insulin resistance, lipids, primary hypertension

## Abstract

**Background:**

Hyperinsulinemia and insulin resistance occurs in obese patients with primary hypertension independent of diabetes and obesity. This study was aimed at assessing serum fasting insulin levels, the homeostatic model assessment for insulin resistance (HOMA-IR), and serum lipid levels in non-obese patients with primary hypertension when compared to normotensive subjects.

**Methods:**

This observational study comprised 100 patients over 18 years of age, divided into two groups. The hypertensive group comprised non-obese patients with primary hypertension (*n*=50); the normotensive group comprised normotensive age- and sex-matched individuals (*n*=50). Patients with diabetes, impaired fasting glucose, obesity, and other causative factors of insulin resistance were excluded from the study. Serum fasting insulin levels and fasting lipid profiles were measured, and insulin resistance was calculated using HOMA-IR. These data were compared between the two groups. Pearson’s correlation coefficient was used to assess the extent of a linear relationship between HOMA-IR and to evaluate the association between HOMA-IR and systolic and diastolic blood pressures.

**Results:**

Mean serum fasting insulin levels (mIU/L), mean HOMA-IR values, and fasting triglyceride levels (mg/dL) were significantly higher in the hypertensive versus normotensive patients (10.32 versus 6.46, *P*<0.001; 1.35 versus 0.84, *P*<0.001; 113.70 versus 97.04, *P*=0.005, respectively). The HOMA-IR levels were associated with systolic blood pressure (*r* value 0.764, *P*=0.0005).

**Conclusion:**

We observed significantly higher fasting insulin levels, serum triglyceride levels, and HOMA-IR reflecting hyperinsulinemia and possibly an insulin-resistant state among primary hypertension patients with no other causally linked factors for insulin resistance. We observed a significant correlation between systolic blood pressure and HOMA-IR.

## INTRODUCTION

Hypertension (HT) is a major public health problem in India, with an estimated disease burden affecting 200 million persons.^1^ Globally the projected burden of HT by 2025 is 1.56 billion people. Overall prevalence of HT in India is 29.8%, with a higher urban prevalence (33.8%) than in rural areas (27.6%).^1^ According to a 2017 report of the Non-Communicable Disease (NCD) Risk Factor Collaboration, HT is the most important cause of mortality and morbidity in India.^2^ Insulin resistance (IR) is a hallmark feature of diabetes and obesity and a key component connecting all aspects of metabolic syndrome. Newer insights in understanding the pathogenesis of primary HT have identified IR as a risk factor for incident HT. The Indian population tends to be more insulin-resistant compared to their white counterparts, attributable to a rapidly increasing body mass index (BMI) and central obesity.^3^ Insulin resistance is defined as decreased or impaired sensitivity to the effects of insulin on the target organs resulting in impaired glucose utilization.^4^ Due to the reduced response of the peripheral tissues to normal physiological levels of insulin, compensatory hyperinsulinemia occurs. Hence hyperinsulinemia is considered a marker of IR and impaired glucose metabolism.^5^ In a study by Ray et al. in India, 35% of the HT patients admitted for acute coronary syndrome had IR.^6^ The Asian Indian phenotype is characterized by a normal BMI and increased abdominal obesity, which explains the high prevalence of IR among Indians.^3^

Insulin resistance causes HT via several mechanisms, including enhanced renal sodium absorption, renin angiotensin system (RAS) activation, augmentation of sympathetic nervous system activity, endothelial dysfunction, and increased peripheral and renal vascular resistance.^7^–^13^ Nakamura et al. observed a blunting of insulin-mediated glucose uptake in adipose tissue via insulin receptor substrate 1 (IRS1), whereas insulin-mediated sodium reabsorption in the proximal tubule was preserved via insulin receptor substrate 2. Hence compensatory hyperinsulinemia in IR can lead to enhanced sodium reabsorption resulting in HT.^14^ Similarly, activation of the RAS results in increased angiotensin II (AT-II), which is a potent vasoconstrictor; AT-II inhibits differentiation of adipocytes, thereby causing IR and mitochondrial dysfunction.^15^ Angiotensin II inhibits the phosphatidyl inositol-3 kinase pathway, thereby affecting downstream insulin-mediated actions. Eventually AT-II decreases insulin-mediated glucose uptake in skeletal muscle, and a proinflammatory state sets in, causing IR. Hypertension and IR are interlinked, and one perpetuates the other.^16^ Our study looked at non-obese and non-diabetic hypertensive individuals and their fasting insulin levels and homeostatic model assessment for insulin resistance (HOMA-IR), and compared them to a normotensive group with the aim of assessing hyperinsulinemia in primary HT.

The primary objective of our study was aimed at comparing the serum fasting insulin levels and calculated HOMA-IR between primary HT and normotensive subjects. The secondary objective was to compare the fasting lipid profile between the two groups. We also sought to assess the linear relationship between HOMA-IR and blood pressure among hypertensive patients.

## MATERIALS AND METHODS

This was an observational study conducted for a period of 18 months in 2015–2016 at a tertiary care center in South India. Study subjects were recruited from the out-patient HT clinic of a general medicine department. Study subjects were enrolled in two groups: the hypertensive group comprised patients >18 years of age who were either treatment-naive hypertensives or known hypertensives (on amlodipine or enalapril, or both). The normotensive group comprised non-HT age- and sex-matched patients. Exclusion criteria for the hypertensive and normotensive groups were: (i) age ≥60 years; (ii) history of diabetes mellitus; impaired fasting glucose, or glucose tolerance; (iii) hypothyroidism; (iv) polycystic ovarian disease; (v) those who had fasting blood sugar 100 mg/dL or more, triglycerides (TGL) >150 mg/dL during screening; (vi) BMI more than 25 kg/m^2^; waist–hip ratio >0.9 in men, >0.85 in women; (vii) pregnant women; (viii) patients diagnosed with secondary HT; (ix) being a chronic or current smoker or alcoholic; and (x) use of beta-blockers and/or thiazide diuretics.

Patients visiting a diabetes and HT clinic once a week as an out-patient service were initially screened for anthropometric criteria by one of the investigators. Consecutive hypertensive patients who satisfied anthropometric criteria were enrolled in this study. Subjects with age, gender, and BMI matching the hypertensive group were selected from a general medicine out-patient clinic. The normotensive group was selected following the same anthropometric criteria and exclusion criteria of the hypertensive group to ensure comparable metabolic and anthropometric variables between the two groups. Informed consent was obtained from all patients who were enrolled in the study. The study was approved by the Institutional Ethics Committee and complied with ethical standards for human subjects as well as the Helsinki declaration (approval number: CSP-MED/14/SEP/18/150). A total of 100 subjects who satisfied the inclusion criteria were enrolled (hypertensive group, *n*=50; normotensive group, *n*=50).

The baseline characteristics of the hypertensive group, including duration of HT, medication history, previous intercurrent illness and additional co-morbidities, anthropometric measurements (height in centimeters, weight in kilograms, BMI, waist–hip ratio) were recorded according to standard procedure, as was the BMI. Waist circumference was measured to the nearest 0.1 cm using a measuring tape midway between lower part of lowest rib and the top of iliac crest, in standing position with arms relaxed at the end of normal expiration. Hip circumference was measured to the nearest 0.1 cm at the level of the greater trochanter.

Laboratory tests included fasting blood sugar (mg/dL), postprandial blood sugar (mg/dL), fasting lipid profile to measure total cholesterol, low-density lipoprotein cholesterol, TGL, high-density lipoprotein cholesterol, and serum fasting insulin levels (mIU/mL). The HOMA-IR, which required measurements of serum fasting insulin levels and fasting glucose, was used to assess IR. The HOMA-IR was calculated as follows: HOMA-IR=fasting insulin (mIU/mL)×fasting blood glucose (mg/dL)/405.

## DATA ANALYSIS

Fasting insulin levels, HOMA-IR values, and lipid parameters were compared between the two groups. The degree of association between either systolic blood pressures or diastolic blood pressures and HOMA-IR was analyzed with Pearson’s correlation coefficient. Descriptive results were explained using tables, continuous variables were expressed as mean±standard deviation, and discrete variables were expressed as number (%). Paired *t* test, chi-square test, and analysis of variance (ANOVA) were used as appropriate to determine the differences between the hypertensive and normotensive groups for the variables (age, BMI, alcoholism, smoking, family history of HT and diabetes, antihypertensive usage, lipid parameters, serum insulin levels and HOMA-IR). A *P* value <0.05 was considered statistically significant. Analysis was done using SPSS software version 17.

## RESULTS

The study had a total of 100 participants, who were divided into two groups based on the presence (hypertensive group, *n*=50) or absence (normotensive group, *n*=59) of HT. The majority of study participants were aged 41–50 years (45%) in both groups; only 6% (*n*=3) of the patients were under 35 years of age.

The study population was sex-matched, with 25 males and 25 females in each group. The baseline characteristics of all participants are shown in Table 1. Mean HT duration in the hypertensive group was 3.66 years (mean duration among male and female patients was 4.06 years and 3.26 years, respectively). The majority of patients in the hypertensive group were receiving amlodipine (70%, *n*=35), with 6% (*n*=3) receiving enalapril, and 24% (*n*=12) receiving both (Table 2). The observed mean serum fasting insulin level (10.97±3.31 mIU/L) was relatively higher among patients taking amlodipine alone compared to those receiving enalapril alone or both amlodipine and enalapril.

**Table 1:** Baseline Characteristics of Study Participants.

Characteristics	Hypertensive Group (*n*=50)	Normotensive Group (*n*=50)
Age (years)	44.50±6.60	44.45±7.80
Male (*n*)	25	25
Female (*n*)	25	25
BMI (kg/m^2^)	21.79±1.48	21.44±2.49
Height (cm)	167.74±6.39	167.78±8.21
Weight (kg)	61.80±6.52	61.05±9.30
Waist–hip ratio (WHR)	0.75±0.04	0.75±0.05
Family history of diabetes mellitus (%)	34%	30%
Family history of HT (%)	38%	26%
Alcoholism in past (%)	12%	10%
Systolic blood pressure (mmHg)	136.00±7.26	111.60±8.56
Diastolic blood pressure (mmHg)	82.50±5.90	71.98±6.25
Fasting blood sugar (mg/dL)	91.14±5.28	89.00±6.68
Postprandial blood sugar (mg/dL)	112.66±13.19	105.22±12.61

Continuous variables expressed as mean±SD; categorical variables in number and percentage.

**Table 2:** Serum Fasting Insulin, Triglyceride (TGL) and High-density Lipoprotein (HDL) Cholesterol Levels Among the Hypertensive Group (*n*=50) Based on Antihypertensive Drug Use.

Parameter	Amlodipine (*n*=35)	Enalapril (*n*=3)	Amlodipine and Enalapril (*n*=12)
Fasting Insulin levels (mIU/L)	10.98±3.31	7.70±2.82	9.19±2.20
TGL (mg/dL)	114.69±26.73	115.33±30.04	110.42±31.94
HDL (mg/dL)	43.17±7.41	42.67±3.51	42.83±8.61

Table 3 shows a comparison of HOMA-IR, fasting insulin levels, and lipid profile between the hypertensive and normotensive groups. Mean HOMA-IR and mean insulin levels were higher in hypertensive patients compared to normotensive patients (both *P*<0.001) (Table 3). Subanalysis for males and females between the two groups separately showed similar results. No significant differences were found when comparing fasting insulin levels between males and females (hypertensives, *P*=0.69; normotensives *P*=0.42), and similarly for HOMA-IR (*P*=0.61 and *P*=0.35, respectively).

**Table 3:** Comparison of HOMA-IR, Fasting Insulin Levels, and Lipid Profile in the Hypertensive and Normotensive Groups.

Parameter	Hypertensive Group (*n*=50)	Normotensive Group (*n*=50)	*P* value
HOMA-IR			
Male	1.38±0.33	0.88±0.29	<0.001
Female	1.32±0.47	0.80±0.30	<0.001
Total	1.35±0.40	0.84±0.30	

Fasting Insulin Levels (mIU/L)			
Male	10.52±2.52	6.75±2.62	<0.001
Female	10.16±3.75	6.16±2.46	<0.001
Total	10.34±3.13	6.45±2.54	

TGL (mg/dL)	113.70±27.66	97.04±30.17	0.005

Total cholesterol (mg/dL)	173.64±30.38	161.44±36.51	0.07

LDL (mg/dL)	110.80±27.82	107.04±36.15	0.56

HDL (mg/dL)	43.06±7.44	43.58±9.24	0.75

HDL, high-density lipoprotein; HOMA-IR, homeostatic model assessment for insulin resistance; LDL, low-density lipoprotein; TGL, tryiglycerides.

Mean HOMA-IR and mean insulin levels were higher in hypertensive patients compared to normotensive patients (both *P*<0.001) (Table 3). Subanalysis for males and females apart showed similar results. Of the lipids, only TGL levels were higher in the hypertensive group compared to the normotensive group (*P*<0.001).

**Figure 1:**
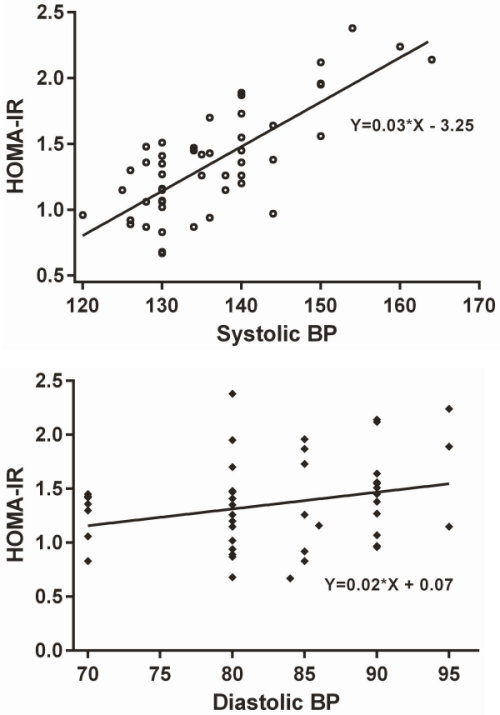
Pearson Correlation between Systolic Blood Pressure and HOMA-IR (*r*=0.764), and between Diastolic Blood Pressure and HOMA-IR (*r*=0.274).

Among hypertensive patients, we observed a significant positive linear relationship between systolic blood pressure and HOMA-IR (*r*=0.764; *P*=0.0005) (Figure 1). Correlation between diastolic blood pressure and HOMA-IR was not significant in our study (*r*=0.274; *P*=0.054).

## DISCUSSION

The aim of this study was to evaluate fasting insulin levels and HOMA-IR in hypertensive patients without other causes for IR. Most of the published data on IR and HT included overweight and/or obese individuals (BMI>25 kg/m^2^) as part of the study groups.^17^,^18^ Our study excluded patients with a BMI> 25 kg/m^2^; the mean BMI in our hypertensive group was 21.79 kg/m^2^. The hyper-insulinemic euglycemic clamp technique is considered the gold standard test and offers a direct measure of IR, but its use in clinical practice is limited.^19^ The HOMA-IR is a surrogate, indirect, validated marker of IR;^20^ it reflects mainly hepatic IR and does not account for the total effect of IR.^21^ The IR cut-off level for this model varies widely in published data, mainly due to the selection criteria of study participants. Singh et al. reported a HOMA-IR cut-off value of 2.5 as an indicator of IR in urban Indian adolescents for identifying metabolic syndrome with a sensitivity of 70% and specificity of 60%.^22^ When comparing the hypertensive group with the normotensive group, we observed significant differences in fasting insulin levels, HOMA-IR, and TGL. A study by Esteghamati et al. showed that hypertensive patients without diabetes had significantly higher fasting plasma insulin levels (9.24 versus 7.86 mIU/L) and higher HOMA-IR (2.16 versus 1.75) when compared to normotensive non-diabetic patients.^17^ Akande et al. observed a 31% IR prevalence among hypertensive Nigerian patients, with a HOMA-IR cut-off value of >3.8 considered to be insulin-resistant.^18^ Although diabetics were excluded, these studies included obese individuals, which explains the higher HOMA-IR value when compared to our study. Patients in a study by Esteghamati et al. had a mean BMI of 28.35 kg/m^2^ in their hypertensive group, and Akande et al. observed obesity among 31% of their hypertensive patients (mean BMI 27.4 kg/m^2^).^17^,^18^

In the CRISPS2 study, a large normotensive group (*n*=1344; mean BMI 23.5; HOMA-IR 1.0) was followed for a mean of 6.4 years; 16% (*n*=84 out of 1344) subsequently developed HT, and the observed values in this incident hypertensive group (mean BMI 25 kg/m^2^, TGL 124 mg/dL, HOMA-IR 1.3, fasting plasma insulin 5.4 mIU/L) were statistically significant.^23^ Our results, when comparing hypertensive to normotensive subjects, were closely in line with the CRISPS2 study with regard to fasting insulin level, HOMA-IR, and TGL. The HOMA-IR was significantly correlated with systolic blood pressure in our study. Dalai et al. observed a significant correlation with serum fasting insulin levels and different HT categories.^24^ These findings support the idea that HT is an insulin-resistant state and hyperinsulinemia and IR can occur among hypertensive patients independent of diabetes, obesity, or hypertriglyceridemia.

Insulin resistance has already been found to be an independent risk factor for incident HT.^25^ It is the main characteristic and core component in metabolic syndrome. Diabetic dyslipidemia attributed to IR is characterized by hypertriglyceridemia, increased low-density lipoprotein cholesterol, and low high-density lipoprotein cholesterol concentrations. The pattern of lipid profile abnormality in primary HT is not well established. In order to assess the variation in lipid parameters in HT, the current study excluded diabetes, obesity, and use of drugs such as beta-blockers and thiazide diuretics to control blood pressure, since both these drugs have been known to increase TGL levels.^26^ We found that TGL was significantly higher in the hypertensive group compared to normotensive controls. Although Esteghamati et al. observed high TGL values (143 mg/dL) among their hypertensive group, their findings were not statistically significant, most likely due to the inclusion of obese patients in both the hypertensive and normotensive groups.^17^ Imazu et al. observed TGL levels less than 150 mg/dL in their hypertensive group.^27^

Angiotensin II (AT-II) can cause either IR in hypertensive individuals or incident HT among those who are already insulin-resistant, hence angiotensin receptor blockers are a better choice compared to calcium channel blockers for blood pressure control among non-diabetic patients with HT. Grosskopf et al. observed that calcium channel blockers (nifedipine) had a positive effect on insulin sensitivity in an elderly age group (70–75 years) but did not have similar effects in people <45 years.^28^

The strength of our study is in the exclusion of secondary causative factors of IR, notably diabetes and obesity among hypertensive patients. By comparison, the added strength of our control enabled comparison for assessing the significant increase in measured fasting insulin levels, calculated HOMA-IR, and lipid profile values attributable due to primary HT.

### Limitations of Our Study

The small sample size in this study allowed only a limited comparison of IR variables in the hypertensive group. Our study did not assess the association between blood pressure control (controlled versus uncontrolled) and IR, since the hypertensive group was not divided into a subgroup of controlled versus uncontrolled HT; nor was the effect of duration of HT on IR studied. In addition, the physical activity of the two groups was not taken into consideration. A prospective study conducted in a large patient cohort is needed to determine the effect of HT and IR, the results of which could potentially change the perspective of practitioners in the pharmaceutical management of hypertensive patients without diabetes.

### Future Research

Prospective studies on non-diabetic normotensive subjects with IR are required to investigate the claim that IR is a risk factor for newly diagnosed HT.

## CONCLUSION

Our study demonstrated that serum fasting insulin levels and HOMA-IR values were higher in patients with primary HT when compared with normotensive controls. Among the lipid parameters, TGL levels were higher in HT patients than in normotensive patients. Systolic blood pressure was significantly correlated with HOMA-IR. After excluding other causes of IR, our observations among hypertensive patients support the finding that HT is an insulin-resistant state.

## Abbreviations

AT-II: angiotensin II
BMI: body mass index
HOMA-IR: homeostatic model assessment for insulin resistance
HT: hypertension
IR: insulin resistance
LDL: low-density lipoprotein cholesterol
NCD: non-communicable disease
RAS: renin angiotensin system
TC: total cholesterol
TGL: triglyceride(s)
